# Kinetic analysis of D-Alanine upon oral intake in humans

**DOI:** 10.1007/s00726-024-03421-6

**Published:** 2024-10-14

**Authors:** Tomonori Kimura, Shinsuke Sakai, Masaru Horio, Shiro Takahara, Shoto Ishigo, Maiko Nakane, Eiichi Negishi, Hiroshi Imoto, Masashi Mita, Kenji Hamase, Yoko Higa-Maekawa, Yoichi Kakuta, Masayuki Mizui, Yoshitaka Isaka

**Affiliations:** 1 Department of Nephrology, Osaka University Graduate School of Medicine, Osaka, Japan; 2 Department of Nephrology, Kansai Medical Hospital, Osaka, Japan; 3 Kansai Medical Clinic for Renal Transplantation, Osaka, Japan; 4 KAGAMI INC, Osaka, Japan; 5 Department of Drug Discovery and Evolution, Graduate School of Pharmaceutical Sciences, Kyushu University, Fukuoka, Japan; 6 Department of Urology, Osaka University Graduate School of Medicine, Osaka, Japan

**Keywords:** D-Alanine, Kinetics, Urinary excretion, Clearance, Distribution, Pharmacokinetics

## Abstract

D-Alanine, a rare enantiomer of alanine, can potentially alleviate the worsening of viral infections and maintain circadian rhythm. This study aimed to analyze the kinetics of D-Alanine upon oral intake. Five healthy volunteers were administered D-Alanine as a single oral dose at 11,236 or 33,708 µmoL (1–3 g). Upon intake of the lower dose, the plasma level of D-Alanine reached its peak concentration of 588.4 ± 40.9 µM with a peak time of 0.60 ± 0.06 h. The compartment model estimated the clearance of D-Alanine at 12.5 ± 0.3 L/h, or 208 ± 5 mL/min, distribution volume of 8.3 ± 0.7 L and half-life of 0.46 ± 0.04 h, suggesting a rapid clearance of D-Alanine. The peak concentration and area under the curve increased proportionally upon intake of the higher dose, while the clearance, distribution volume and half-life did not. The urinary ratio of D-Alanine per sum of D- and L-Alanine reached its peak of nearly 100%, followed by a slow decline. The peak time of the urinary ratio was 1.15 ± 0.15 h, showing a time lag of blood to urine excretion. Fractional excretion, a ratio of the clearance of a substance per a standard molecule in kidney, of D-Alanine increased from 14.0 ± 5.8% to 64.5 ± 10.3%; the latter corresponded to the urinary clearance of D-Alanine as about 77 mL/min for an adult, with a peak time of 1.90 ± 0.56 h. D-Alanine was quickly absorbed and appeared in blood, followed by urinary excretion. This kinetic analysis increases our fundamental knowledge of the oral intake of D-Alanine for the chronic dosing.

Trial number: #UMIN000050865.

Date of registration: 2023/6/30.

**Supplementary Information:**

The online version contains supplementary material available at 10.1007/s00726-024-03421-6.

## Introduction

D-Alanine is a natural nutrient. D-Amino acids, including D-Alanine, are the enantiomers of L-amino acids that are dominant in life. Recent studies revealed that D-Alanine is present in mammals, including humans (Armstrong et al. 1991; Kimura et al. 2016; Nagata et al. 1987), and is connected to many physiological functions and diseases (Kimura et al. 2023; Lee et al. 2020). The blood level of D-Alanine decreases in severe viral infections such as COVID-19 or influenza virus infection (Kimura-Ohba et al. 2022, 2023), while it increases in patients with kidney diseases (Hesaka et al. 2019; Kimura et al. 2016). D-Alanine can serve as a co-agonist of the *N*-methyl-D-aspartate (NMDA) receptor (Kleckner and Dingledine 1988) and activate macrophages (Suzuki et al. 2021). The protective effect of D-Alanine against diseases has also been demonstrated in rodent models. These include viral infections such as COVID-19 and influenza viral infection (Kimura-Ohba et al. 2022), experimental colitis (Umeda et al. 2023) and acute kidney injury (Iwata et al. 2022). D-Alanine also has a close association with the circadian rhythm (Karakawa et al. 2013; Miyoshi et al. 2009; Sakai et al. 2024). Meanwhile, D-Alanine has a clear circadian rhythm (Morikawa et al. 2008) and maintains this rhythm in a rodent model (Sakai et al. 2024). Through the regulation of circadian rhythm, D-Alanine maintains physical conditions, such as sleep and activity, glucose production, and potentially immune responses (Sakai et al. 2024).

About less than a few % of alanine in blood is D-Alanine, while the rest is L-Alanine (Hesaka et al. 2019; Kimura et al. 2016). D-Alanine is not synthesized in mammalian cells. Therefore, D-Alanine in mammals is of external origins, such as food and the intestinal microbiome (Sasabe et al. 2016). D-Alanine is relatively rich in fermented foods or fish from brackish water (Bruckner et al. 1995; Eto et al. 2011; Gogami et al. 2011; Miyoshi et al. 2014). D-Alanine in mice is also derived from the intestinal microbiome (Gonda et al. 2023; Lee et al. 2022; Morikawa et al. 2007; Ota et al. 2014; Qiu et al. 2023; Sasabe et al. 2016). D- and L-Alanine taste different, with the former tasting sweeter (Schiffman et al. 1981). D, L-Alanine, a form that includes both L- and D-enantiomer, has been approved as a food additive in the world including Japan and United States, and is primarily used as a seasoning.

D-Alanine can correct the disturbed circadian cycle and may have a therapeutic potential for many life style-related diseases; however, much is still not known about its mechanism of action. This includes the kinetics of D-Alanine in the body (Kimura et al. 2023). After oral intake and intestinal absorption, D-Alanine enters the blood and is delivered to the tissues. About 20% of cardiac output is delivered to the kidney, where the blood is subjected to glomerular filtration. After filtration, about 80% of D-Alanine is reabsorbed at the proximal tubules, whereas the rest is excreted into the urine (Hesaka et al. 2019; Sakai et al. 2024). The reabsorbed fraction of D-Alanine is oxidized by D-Amino acid oxidase (DAO), which is predominantly present in the proximal tubules (Koga et al. 2017; Konno and Yasumura 1983; Krebs 1935), and this reaction results in the production of peroxide and pyruvate (Sakai et al. 2024). D-Alanine in the blood is delivered to several tissues. Thus far, it has been detected in endocrine tissue, such as the pancreatic islets, the adrenal glands, and the pituitary gland (Morikawa et al. 2007), as well as the brain, liver and kidney (Miyoshi et al. 2009).

Urinary excretion and oxidation by DAO contribute to maintaining blood D-Alanine levels. The level of D-Alanine increases up to 82 µM in patients with kidney diseases (Hesaka et al. 2019; Kimura et al. 2016, 2023) or 100 µM in *Dao*-deficient rodents (Gonda et al. 2023; Karakawa et al. 2013; Miyoshi et al. 2009). D-Alanine is not incorporated in proteins by translation, and other biological processes that clear D-Alanine have not been found. Therefore, this study aimed to analyze the basic kinetics of D-Alanine in the body, using general kinetic analysis.

## Methods

### Study design and participants

This is an open, non-randomized study. Participants were healthy adults aged ≥ 20 or older who took no medication in the previous month. We recruited 5 volunteers from three centers in Japan between July 2023 and December 2023. Subjects were instructed to avoid fermented food from the day before until the end of the study. Subjects fasted after their evening meal for 10 h until the first blood draw. At 8 am, each received a packaged powder of D-Alanine (Direct Alanine^@^, KAGAMI INC) with 200 mL of water. Blood samples (100 µL) were taken in EDTA-2 K tubes before administration, and 0.25, 0.5, 0.75, 1, 2, 4, 8, and 24 h thereafter. Blood samples were immediately placed on ice water. Within 8 h of blood collection, plasma was collected by centrifugation at 830 x g for 15 min at 4 °C. Urine samples were taken after each blood sampling. Previously, 0.2 g/kg of D-Alanine was treated in mice infected with Covid-19 (Kimura-Ohba et al. 2022). This dose corresponds to 10 g in human weighted 50 kg. Taking the safety margin sufficiently, the test was conducted with different volumes: 1 g [11,236 µmoL] or 3 g [33,708 µmoL] of D-Alanine. Each study was conducted with an interval of at least one week. Followings are the specification of Direct Alanine^@^ used in this study: amount, 2.0 ± 0.10 g per a package; alanine content, 99.0–103%; D-Alanine composition ratio, 49.0–51.0%. Upon sampling inspection, the amount was measured using electron balance whereas alanine content and D-Alanine composition ratio were quantified using two-dimensional high-performance liquid chromatography (2D-HPLC) as follows. This study was conducted in compliance with the Declaration of Helsinki, the Ethical Guidelines for Medical Research Involving Human Subjects. This study was registered in UMIN-CTR (#UMIN000050865). Approval for all facilities was obtained from the Central Ethics Review Committee of Osaka University (#122472). Written informed consent was obtained from all the participants.

### Quantification of D-Amino acids

Sample preparations and quantification of amino acid enantiomers by a 2D-HPLC system were performed as previously described (Hamase et al. 2010, 2018). This system can detect amino acid enantiomers ranging from ca. 1 fmol to 100 pmol quantitatively with chiral selectivity without severe interference from intrinsic substances. The relative standard deviations of between-run precision were 1.10–8.19% with a high performance of reproducibility (Hamase et al. 2010, 2018; Kimura et al. 2016). Followings are the methods in brief. Twenty-fold volumes of methanol were added to the sample and an aliquot (10 µL of the supernatant obtained from the methanol homogenate) was placed in a brown tube. After drying the solution under reduced pressure, 20 µL of 200 mM sodium borate buffer (pH 8.0) and 5 µL of fluorescence labeling reagent (40 mM 4-fluoro-7-nitro-2,1,3-benzoxadiazole in anhydrous acetonitrile) were added and then heated at 60 °C for 2 min. An aqueous solution of 0.1% (v/v) trifluoroacetic acid (75 µL) was added, and 2 µL of the reaction mixture was subjected to the 2D-HPLC.

The enantiomers of the amino acids were quantified using the 2D-HPLC platform. The fluorescence-labeled amino acids were separated using a reversed-phase column (Singularity RP column, 1.0 mm i.d. × 50 mm; provided by KAGAMI Inc., Osaka, Japan), with the gradient elution using aqueous mobile phases containing acetonitrile and formic acid. To determine D- and L-amino acids separately, the fractions of amino acids were automatically collected using a multi-loop valve and transferred to the enantioselective column (Singularity CSP-001 S, 1.5 mm i.d. × 75 mm; KAGAMI Inc.). The mobile phases are the mixed solution of methanol- acetonitrile containing formic acid, and the fluorescence detection was carried out at 530 nm with excitation at 470 nm using two photomultiplier tubes.

Target peaks were quantified by scaling the standard peak shape.^19^ In this method, the shape of a peak was used for the identification of the substrate, whereas the magnitude of the intensity was used for quantification. From the chromatogram of a sample, target shapes of amino acid enantiomers were identified based on the elution time and shape of the peak. The peak shape obtained by the standard amino acid enantiomer was superimposed to the obtained peak sections, and the magnification constant best fitted to the target peak was identified. The concentration of the target enantiomer was calculated by using an identified magnification constant and the calibration lines. The peak shape method potentiated quantification within a few seconds. The fully-automatic 2D-HPLC system required < 10 min for the measurements of each D-Amino acid, including separation, identification, and quantification steps. The D-Amino acid ratio was defined as the percentage (%) of D-Amino acids to the sum of L- and D-Amino acids.

### Fractional excretion (FE)

Fractional excretion (FE) is the ratio of the clearance of a substance to the clearance of a standard molecule. The FE was calculated as follows: Ux (mg/dL) × Sy (mg/dL)/Sx (mg/dL) × Uy (mg/dL), where x is a substrate substance, y is a reference substance, Ux is the urinary concentration of x, Sy is the plasma concentration of y, Sx is the plasma concentration of x, and Uy is the urinary concentration of y. As a reference, we used D-Asparagine and D-Serine based on close dynamics to inulin, a gold standard for measuring kidney function (Kawamura et al. 2022; Taniguchi et al. 2023).

### Kinetic study

Time courses of D-Alanine plasma concentrations were analyzed by noncompartmental and compartmental methods. The area under the curve from zero to the end of the observation period was estimated using the log-linear trapezoidal rule. The selection of the end of the observation, not infinite, is based on the concept that despite the low concentration, the presence of endogenous D-Alanine contradicts the hypothesis of an infinite model that the level of D-Alanine regresses to zero after infinite time.

For the estimation of detailed kinetic parameters, we applied the compartment models, which was possible even in the presence of endogenous substance. Estimation of compartmental parameters were performed for each individual after fitting the data to one- or two-compartment models. The discrimination of the models was based on the goodness-of-fit comparisons using the CV of parameter estimates and Akaike’s Information Criterion. The simple unweighted results are shown after observing no improvement in the various weighting schemes. Analyses were performed using WinNonlin Professional (version 8.0) software (Pharsight, Cary, NC).

### Statistical analysis

Data were expressed as mean ± standard deviation, or as count and ratio (%). A comparison between two groups was performed using the Wilcoxon signed-rank test. Data visualization was performed using GraphPad Prism 8.0 and statistical analyses were performed using Stata 15.0.

## Results

### Kinetics of D-Alanine in blood

Five healthy volunteers were enrolled. They were aged 47 ± 11, 80% in male, and body mass index of 21 ± 2 kg/m^2^. After oral intake of D-Alanine, either at the dose of 12,366 (low dose) or 33,708 µmoL (high dose), blood samples were collected at specific time intervals. The participants went asleep naturally at night. There were no adverse events.

**Fig. 1:**
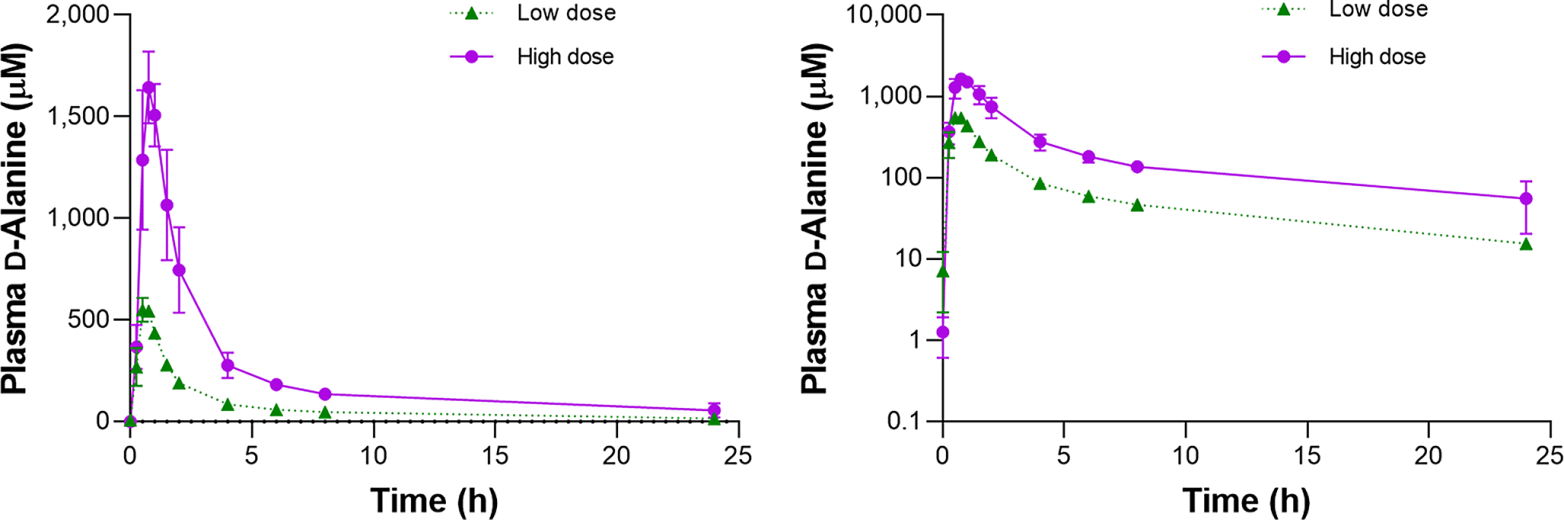
Plasma D-Alanine levels after oral intake of D-Alanine. Y axes with linear (left) or log-scaled (right) are shown

After oral intake of D-Alanine, the level of D-Alanine in the plasma quickly reached its peak concentration (Fig. 1, Supplementary Figures S1 and S2). The kinetic parameters are shown in Table 1. The peak concentration (C_*max*_) was 588.4 ± 40.9 µM for the low dose and 1692.0 ± 69.3 µM for the high dose (*P* < 0.05 for low versus high dose), revealing a clear proportionality with the dose. The t_*max*_, mean time to the peak, was 0.60 ± 0.06 h for low dose and 0.85 ± 0.06 h for high dose. The elimination phase began immediately after the peak, and the slope of the concentration curve was exponential. After 24 h, the plasma level of D-Alanine became closer to the endogenous level. Noncompartmental modeling calculated the AUC for 24 h of 1,649 ± 58 h x µmoL/L for low dose and 4,942 ± 209 h x µmoL/L for high dose (*P* < 0.05), again showing a clear dose-dependent proportionality. Regarding the analysis of L-Alanine, the level of L-Alanine in plasma quickly reached its peak concentration, followed by a sharp decline to a level similar to the baseline, and was relatively constant thereafter (Supplementary Figures S1 and S2). The level of L-Alanine in plasma also has a peak, since the powder used in this study contains identical amount of D- and L-Alanine. The ratio of D-Alanine in plasma, as calculated as the concentration of D-Alanine per sum of L- and D-Alanine, showed a similar kinetic curve as that of D-Alanine with a higher peak for the high dose compared to that for the low dose (*P* < 0.05; Supplementary Figures S1 and S2).

**Table 1:** Kinetic parameters of plasma D-Alanine after oral intake

Dose	C_*0*_	T_*max*_	C_*max*_	AUC_*last*_	Clearance		Distribution	Half life
(µmoL)	(µmoL/L)	(h)	(µmoL/L)	(h x µmoL/L)	(L/h)	(mL/min)	volume (L)	(h)
11,236	2.0 ± 0.4	0.60 ± 0.06	588.4 ± 40.9	1649.0 ± 58.0	12.5 ± 0.3	208 ± 5	8.3 ± 0.7	0.46 ± 0.04
33,708	1.3 ± 0.3^†^	0.85 ± 0.06^†^	1692.0 ± 69.3*	4942.9 ± 208.9*	10.5 ± 0.8*	175 ± 14*	8.9 ± 0.4^†^	0.60 ± 0.06^†^

AUC, area under curve. *P < 0.05, ^†^*P* > 0.05

Despite the low concentration, endogenously present D-Alanine contradicts the hypothesis of the infinite model that the level of D-Alanine regresses to zero after infinite time. Therefore, we abandoned further analysis using a non-compartment model and applied compartment models to estimate further kinetic parameters. One compartment model was selected based on the goodness-of-fit comparisons. In a small dose study, the clearance of D-Alanine was estimated to be 12.5 ± 0.3 L/h, or 208 ± 5 mL/min, the distribution volume as 8.3 ± 0.7 L, and half-time, time required to decrease the level of D-Alanine from the peak in half, as 0.46 ± 0.04 h (Table 1). The analysis for the high dose showed similar estimates (*P* > 0.05; Table 1), except a slightly-reduced clearance (*P* < 0.05). The effects on other D- and L-amino acids were unclear because of the values were wide in range and the peaks were, if present, small (Supplementary Figure S1 and S2).

### Urinary kinetics of D-Alanine

**Fig. 2:**
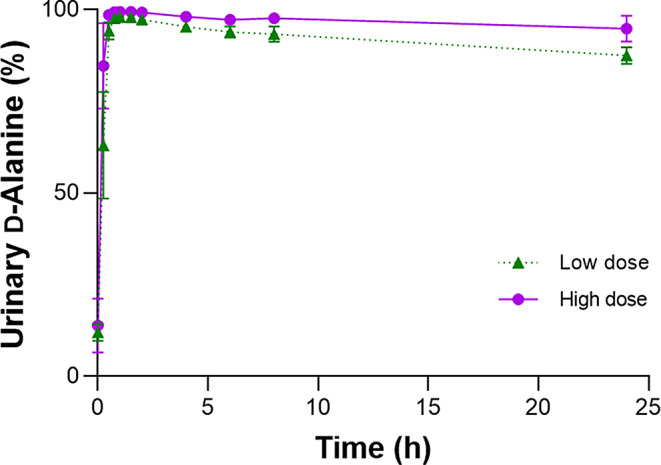
Urinary D-Alanine ratios after oral intake of D-Alanine

The level of D-Alanine in plasma is regulated through urinary excretion, and we examined the urinary excretion kinetics of D-Alanine. We calculated the urinary ratio of D-Alanine per sum of D- and L-Alanine for the analysis. The urinary ratio of D-Alanine was initially 12.0 ± 2.4% and quickly reached nearly 100%, followed by a slow decline (Fig. 2; Table 2 and Supplementary Figure S3). The analysis of the high dose showed a similar trend with a slightly higher peak for high dose (*P* < 0.05; Fig. 2; Table 2 and Supplementary Figure S4). The T_*max*_ was indifferent between groups with 1.15 ± 0.15 h for low dose (*P* > 0.05). The plasma D-Alanine level peaked earlier than the urinary ratio, since D-Alanine enters plasma after oral intake and then enters the urine.

**Table 2:** Urinary excretion kinetics of D-Alanine ratio

Dose	C_*0*_	T_*max*_	C_*max*_
(µmoL)	(%)	(h)	(%)
11,236	12.0 ± 2.4	1.15 ± 0.15	98.2 ± 0.3
33,708	13.9 ± 3.3^†^	1.10 ± 0.10^†^	99.5 ± 0.1*

%, D-Alanine ratio. *P < 0.05, ^†^*P* > 0.05

**Fig. 3:**
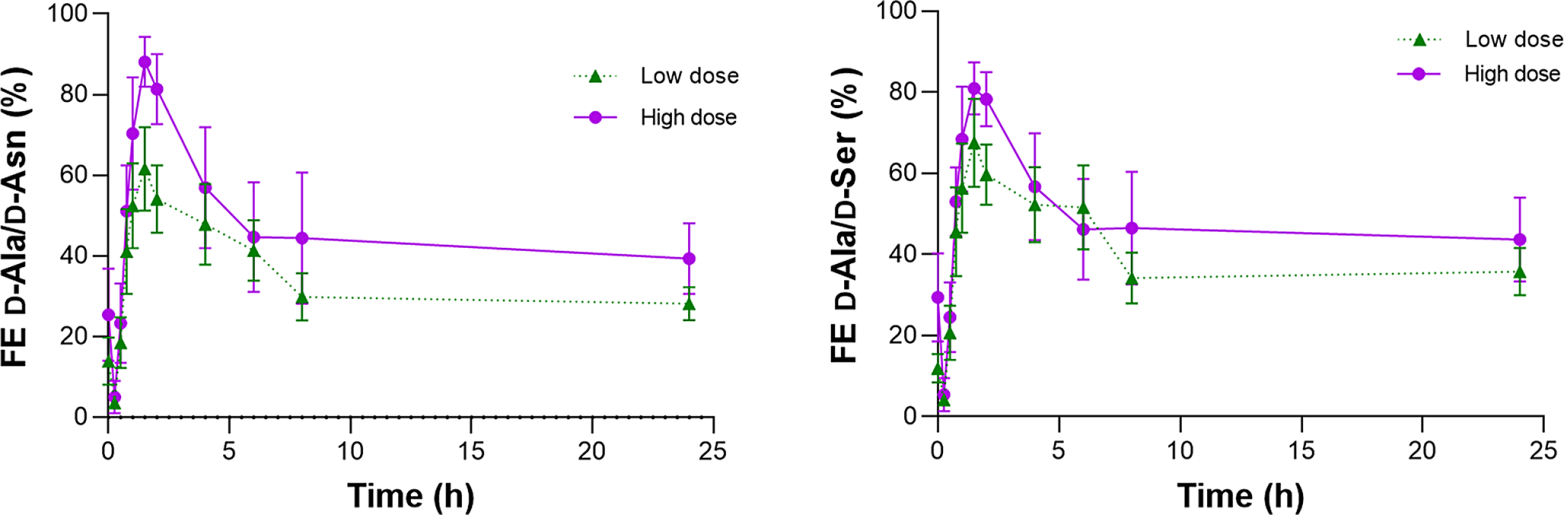
Fractional excretion of D-Alanine after oral intake of D-Alanine. Fractional excretion (FE) was calculated using D-Asparagine or dD-Serine as references

To further delineate the urinary excretion dynamics, we calculated the fractional excretion (FE). FE is the ratio of the clearance of a substance to the clearance of a standard molecule in kidney. It is used to monitor the excretion ratio of the substance after glomerular filtration. We used D-Asparagine or D-Serine as references, as the urinary excretion dynamics are close to the ideal reference, inulin. Fractional excretion (FE) of D-Asparagine and D-Serine are closely to that of inulin, i.e., nearly 100%, whereas a classical marker of creatinine involves a large proportional bias because of high FE value of 150–200% (Kawamura et al. 2022; Taniguchi et al. 2023). The initial D-Asparagine-based FE of D-Alanine (FE D-Ala/D-Asn) was 14.0 ± 5.8% (Fig. 3; Table 3). Just after oral intake of D-Alanine at the low dose, FE D-Ala/D-Asn decreased because of the rapid increase in the blood D-Alanine level. After this, FE D-Ala/D-Asn increased quickly to its peak of 64.5 ± 10.3%, followed by a decline and became constant at about 30%. Suppose the glomerular filtration rate, and its almost identical D-Asparagine clearance, was 120 mL/min; the urinary clearance of D-Alanine at the baseline and the peak and constant stages was estimated to be 16.8, 77.4, and 36.0 mL/min, respectively. It was suggested that the urinary excretion of D-Alanine increases when the blood level increases. The T_*max*_ was 1.90 ± 0.56 h. The initial decline of the FE likely represented the time-lag between the peak concentration of D-Alanine in plasma and urine. The FE of L-Alanine, on the other hand, was constant throughout the study (Supplementary Figure S5). The dynamics of FE D-Ala/D-Asn was close to D-Serine-based FE D-Alanine (FE D-Ala/D-Ser, Fig. 3 and Supplementary Figure S5). The analysis of the high-dose data showed a similar trend, with the C_*max*_ of FE D-Ala/D-Asn being 87.3 ± 4.3% (Fig. 3; Table 3).

**Table 3:** Kinetics of fractional excretion of D-Alanine

Dose	C_*0*_	T_*max*_	C_*max*_
(µmoL)	(%)	(h)	(%)
11,236	14.0 ± 5.8	1.90 ± 0.56	64.5 ± 10.3
33,708	25.4^†^±5.1	1.70 ± 0.12^†^	87.3 ± 4.3^†^

%, fractional excretion of D-Alanine with D-Asparagine as a reference. ^†^*P* > 0.05

## Discussion

This study unraveled the dynamics of D-Alanine in the body after oral intake. Upon oral intake, D-Alanine is quickly absorbed and enters the bloodstream. The plasma level of D-Alanine peaks about 30 min after oral intake, followed by exponentially quick clearance. The blood level reaches close to the endogenous level after 24 h. The D-Alanine that enters the bloodstream is delivered to the kidney and excreted into urine. The peak in urinary level follows the blood level and is reached about 1 h after oral intake, reflecting the time lag between it entering the blood and urinary excretion. The FE of D-Alanine increases upon oral intake, and the significance of urinary clearance as part of whole-body clearance was assessed. This provides key insight into the physiology of D-Alanine.

D-Alanine is efficiently absorbed like L-Alanine in the digestive tract. Orally ingested D-Alanine quickly appears in blood, and the blood level of D-Alanine shows a dose-dependent increase to the same range as L-Alanine. D-Alanine is present in food (Miyoshi et al. 2014), and eating foods rich in D-Alanine can induce an increase in the blood D-Alanine level. In rodent models, D-Alanine is delivered to the glucose metabolism-related tissues, such as the adrenal gland, pituitary gland and pancreas (Etoh et al. 2009; Lee et al. 2022; Miyoshi et al. 2009; Morikawa et al. 2007; Ota et al. 2014; Qiu et al. 2023), suggesting the metabolic role of D-Alanine (Sakai et al. 2024). The amino acid transporters are responsible for the absorption of D-Alanine. Transporters recognize the chirality of the amino acid for chiral-selective transportation (Foster et al. 2016; Hesaka et al. 2019; Kimura et al. 2023; Rosenberg et al. 2013; Wiriyasermkul et al. 2023). The efficacy of the D-Alanine transport system is likely high. Many amino acid transporters remain uncharacterized, especially for D-Amino acids, usually found in trace amounts (Foster et al. 2016; Rosenberg et al. 2013; Wiriyasermkul et al. 2023). In the case of D-Serine, a relatively well-studied D-Amino acid, only five transporters were identified to deliver D-Serine (Kimura et al. 2023; Wiriyasermkul et al. 2023). Currently-uncharacterized transporters are thought to deliver D-Alanine.

The volume of distribution of D-Alanine was estimated to be about 9 L, regardless of the dose. This volume exceeds the plasma volume of ~ 4.6 L, if the body weight is assumed to be 60 kg. Therefore, D-Alanine is distributed outside the plasma and delivered to the tissues. Additionally, the distribution volume is likely much less than that of L-Alanine. Since the tissue distribution is limited and FE is high, D-Alanine in the blood is efficiently excreted into urine. Within the estimated 200 mL/min clearance, urinary excretion is responsible for the 16.8−77.4 mL/min. In the case of L-Alanine, FE is close to 1%, suggesting almost negligible urinary excretion. Despite this, the level of L-Alanine in the blood decreases quickly from the peak and becomes constant after oral uptake. This fact suggests that the distribution volume of L-Alanine is higher than that of D-Alanine, and orally ingested L-Alanine is quickly re-delivered to the tissues.

In response to oral uptake, the urinary clearance of D-Alanine increases. After glomerular filtration, D-Alanine is inefficiently reabsorbed by the proximal tubules, while L-Alanine is almost completely reabsorbed. This is reflected in the higher FE of D-Alanine compared to that of L-Alanine. In an adult with a GFR of 120 mL/min, 77.4 mL/min of D-Alanine in the blood is cleared from the urine at peak, while the proximal tubules absorb the rest (42.6 mL/min). The transporter system for D-Alanine is inefficient in the proximal tubules (Kimura et al. 2023). Despite this, the FE of D-Alanine did not reach 100% when the blood level of D-Alanine increased, and the original urine just after glomerular filtration was assumed to contain much higher level of D-Alanine. Instead of reaching its maximum capacity to reabsorb D-Alanine, the proximal tubules’ transporter system can upregulate the amount reabsorbed.

This study confirmed the importance of kidney and metabolism in the regulation of D-Alanine amount in body. The blood level of D-Alanine increases in patients with kidney disease or *Dao*-deficient rodent. The increase in the blood D-Alanine level in kidney failure is a result of (i) reduced kidney blood flow and glomerular filtration and subsequent urinary excretion and (ii) reduced oxidation of D-Alanine. Between two, the reduced kidney blood flow is the upstream factor that induces an increase in D-Alanine level. In mice lacking DAO activity, the blood level of D-Alanine was elevated to about 100 µM with a striking increase in urinary level (Karakawa et al. 2013; Miyoshi et al. 2009), suggesting the key importance of metabolism in the regulation of D-Alanine in body.

The results of this study provide a key information for the supplementation of D-Alanine for the broad range of diseases. Orally-ingested D-Alanine is essential for homeostasis. During severe viral infection, the level of D-Alanine decreases (Kimura-Ohba et al. 2022, 2023), whereas supplementation of D-Alanine relieves the worsening of viral infections (Kimura-Ohba et al. 2022). D-Alanine mediates signal transduction in cells through the circadian network (Sakai et al. 2024), while D-Alanine also exerts its physiological function through oxidation (Sakai et al. 2024; Suzuki et al. 2021). A reduced level of D-Alanine may suggest an increase in the oxidation of D-Alanine. Through the maintenance of blood D-Alanine level, supplementation of D-Alanine is expected to be protective against diseases (Iwata et al. 2022; Kimura-Ohba et al. 2022; Umeda et al. 2023), leading to the propensity of healthy longevity (Sakai et al. 2024). This profound function of D-Alanine is anticipated in use as a supplement. After the original version of this manuscript was published online, one group demonstrated the potential effect of D-Alanine supplementation on the kidney function of human (Oshima et al. 2024). In agreement with the present study, the level of D-Alanine in blood naturally increased upon intake without major adverse effect. Their observation, the increase in eGFR following the uptake of D-Alanine, remained unclear because the limited number of participants and the insufficient accuracy of eGFR. Besides this, the maintenance of blood D-Alanine level in the long-term could suppress worsening of viral infections and maintain body homeostasis, thus rendering the maintenance of body function including the kidney.

This study had several limitations. The number of participants was limited. Therefore, we could not analyze the effects of basic demographic factors, such as age and sex, on the kinetics of D-Alanine. The clearance of D-Alanine by DAO was not estimated. Despite this, the precise measurement of D-Alanine potentiates a detailed kinetic analysis.

In conclusion, we clarified the kinetics of D-Alanine after oral intake. While D-Alanine is efficiently absorbed after oral intake, D-Alanine is delivered to tissues with a distribution volume larger than the plasma volume, followed by rapid clearance. This kinetic analysis adds to the basic knowledge that is required for the modulation of physiological processes after the oral intake of D-Alanine.

## Electronic supplementary material

Below is the link to the electronic supplementary material.

Supplementary Material 1

## Data Availability

No datasets were generated or analysed during the current study.
