# Supplementary material for: Kinetic analysis of D-Alanine upon oral intake in humans
Source: Amino Acids. 2024 Oct 14;56(1):61. doi: 10.1007/s00726-024-03421-6 (PMC11473621; doi:10.1007/s00726-024-03421-6)
Supplement: Supplementary file 1 — Supplementary Material 1 [file 726_2024_3421_MOESM1_ESM.pdf]

Supplementary Information

Kinetic analysis of D-alanine upon oral intake in humans

This PDF file includes:

Supplementary Figures S1–S5

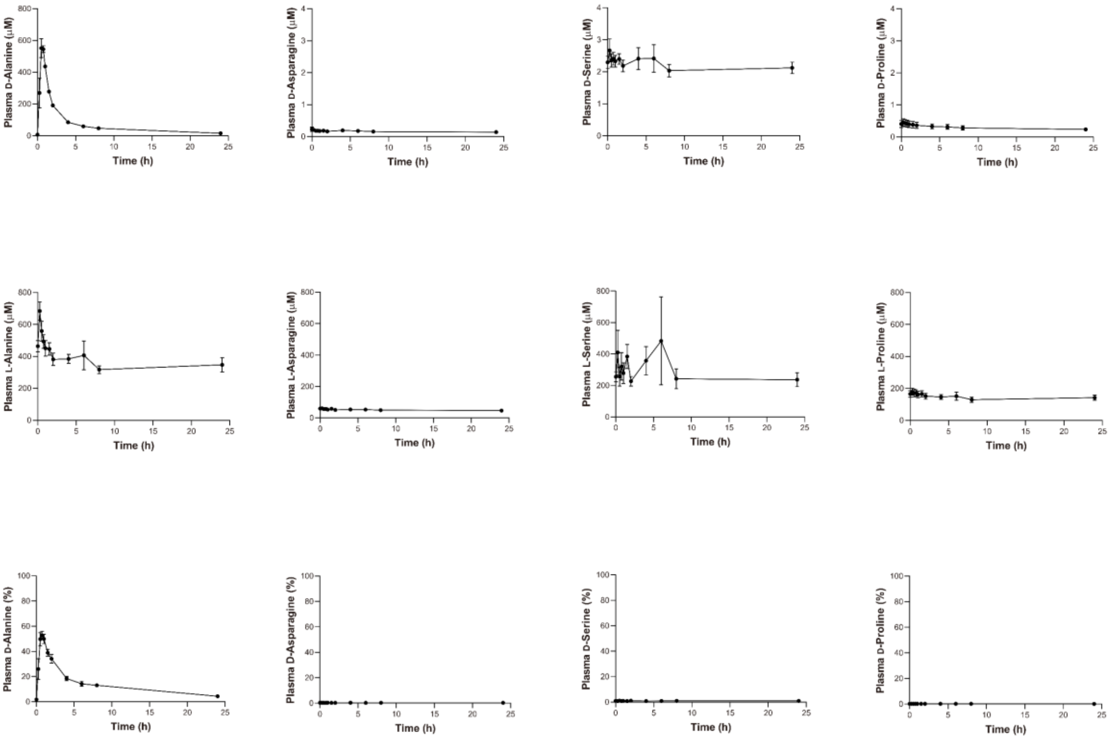

Supplementary Figure S1. Plasma D-amino acid levels or ratios after oral uptake of low dose of D-alanine.

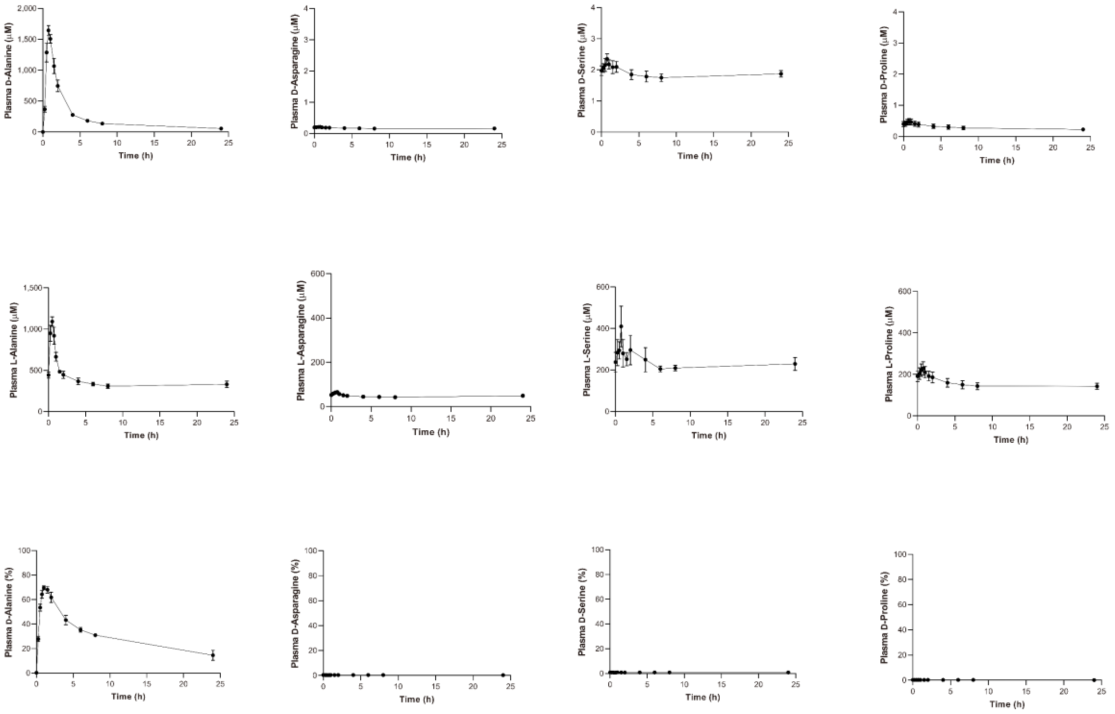

Supplementary Figure S2. Plasma D-amino acid levels or ratios after oral uptake of high dose of D-alanine.

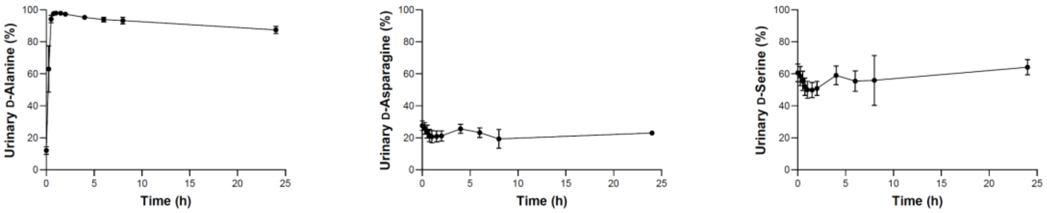

Supplementary Figure S3. Urine D-amino acid levels or ratios after oral uptake of low dose of D-alanine.

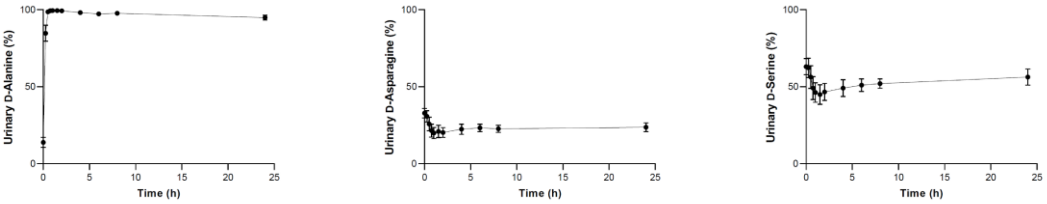

Supplementary Figure S4. Urine D-amino acid levels or ratios after oral uptake of high dose of D-alanine.

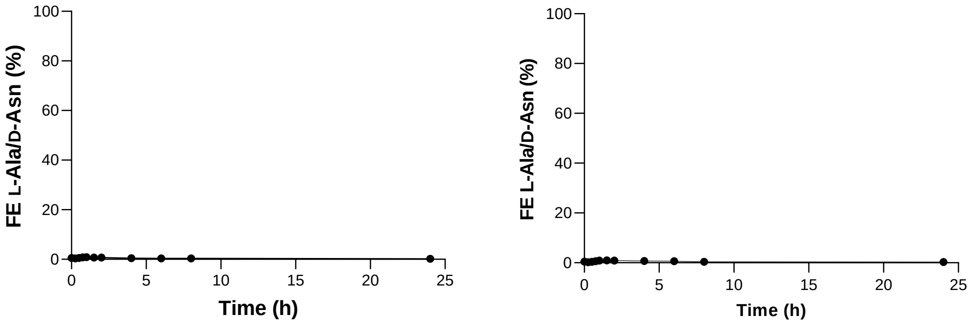

Supplementary Figure 5. Urinary fractional excretion (FE) of L-alanine after oral uptake. (A) Low dose. (B) High dose. FE is calculated using D-asparagine as references.
